# Materia Medica and Therapeutics

**Published:** 1848-06

**Authors:** 


Art. V.—*Materia Medica and Therapeutics:* By Martyn Paine, M.D., Professor of the Institutes of Medicine and Materia Medica in the University of New York, etc, : S. S. & W. Wood, 1848. pp. 411, 8vo.

Dr. Paine's indefatigable pen has produced another book, the objects of which are thus set forth in the preface:

"1. To arrange the *Materia Medica* upon intelligible, physiological and therapeutical principles.

"2. To indicate the relative therapeutic value of the various articles, under their different denominations, by arranging them in the order of their value.

"3. To give to the student a comprehensive and ready view of the merits of the various articles composing the *Materia Medica*, and of their relations to each other, physiologically considered. The *arrangement* itself is also indicative of the pathological and therapeutical principles which lie at its basis. The work was originally designed, therefore, as a *Compendium of Rational Therapeutics.* With these objects in view, the author has now connected the work throughout with his Institutes of Medicine by *references* to the latter, and has introduced a variety of other practical matter which does not occur in his "*Therapeutical Arrangement*," but which seemed conducive to a more amplified work on Therapeutics. It is especially recommended to the student that he should examine the References which are made to the work on the *Institutes of Medicine*, as they may fall incidentally under his notice."

Thus the author himself has to some extent set limits to the circulation of this volume. To the young gentlemen attending his lectures it will prove a convenient and useful work. The usual pharmaceutical statements, and the physical and botanical characteristics of plants, are omitted. A very full index is provided, and this with the table of contents, which is itself a synopsis of the author's arrangment, will make the reference easy. The poetical motto, from Burns, seems to us a little far-fetched and fanciful; and the dedication is certainly more truthful than tasteful. C.

